# Editorial: The psychology of fake news on social media, who falls for it, who shares it, why, and can we help users detect it?

**DOI:** 10.3389/fpsyg.2023.1236748

**Published:** 2023-07-20

**Authors:** David J. Robertson, Mark P. Shephard, Anthony Anderson, Narisong Huhe, David N. Rapp, Jens K. Madsen

**Affiliations:** ^1^School of Psychological Sciences and Health, University of Strathclyde, Glasgow, United Kingdom; ^2^School of Government and Public Policy, University of Strathclyde, Glasgow, United Kingdom; ^3^Department of Psychology, Northwestern University, Evanston, Illinois, IL, United States; ^4^School of Education and Social Policy, Northwestern University, Evanston, IL, United States; ^5^Department of Psychological and Behavioural Science, London School of Economics and Political Science, London, United Kingdom

**Keywords:** fake news, social media, news sharing, misinformation, disinformation and bias

## Introduction

The proliferation of fake news on social media has become a major societal concern which has been shown to impact elections, referenda, and effective public health messaging (Lewandowsky et al., 2017). To combat this, there is now a growing body of research that focuses on the role of psychological and behavioral science in understanding and mitigating the spread of misinformation (Rapp and Salovich, 2018; Van Bavel et al., 2020). For example, research on belief revision has reported a “continued influence effect” (CIE) where misinformation lingers in the mind of a person despite being categorically refuted (e.g., Ecker et al., 2010; Desai et al., 2020), simulations have attempted to replicate the seepage of misinformation in social networks (Lewandowsky et al., 2019), and inoculation theorists are building training tools to understand and enhance psychological resistance against misinformation. Such attempts have been conducted in the context of COVID-19 (Basol et al., 2021), political disinformation (Roozenbeek and van der Linden, 2020), and climate change (Maertens et al., 2020). While it is clear that important advances have been made in our understanding of the critical psychological functions that underpin how individuals seek out, process, and share misinformation—there is still much to do. Therefore, in this special topic, we are delighted to introduce six new papers which present novel, interesting, and engaging contributions to our understanding of the fake news phenomenon.

While investigating the factors that support an individual's ability to detect fake news has taken a prominent position in existing research, there has been less focus on sharing behavior. Understanding the reasons that drive social media users to share fake news will be key to developing effective interventions. In this special issue, papers by t'Serstevens et al., Shephard et al., and Ahmed et al. provide new data on some of the components that may drive the sharing of fake news. Findings from t'Serstevens et al. using a mock Twitter design, show that fake news is more likely to be reacted to and shared, and that self-assessed news veracity and an “activist-type” type behavior play a key role in driving the sharing of misinformation. Both Shephard et al. and Ahmed et al. compliment this research by showing that individual differences in emotional stability, fear of missing out (FOMO), self-regulation, belief in accuracy, and cognitive ability also play important roles in understanding *why* people share fake news and disinformation (using non-partisan content and deepfake video stimuli respectively).

Turning to a focus on the efficacy of news veracity interventions, Bruchmann et al. present timely and important data on the effectiveness of attaching indicators of political bias to news items. However, they find that rather than positively influencing users' understanding of the effect of such bias on the news presented to them, such indicators may inadvertently increase exposure to biased news sources, making people more likely to confirm their previous worldview, and more prone to the seeking of bias in future news choices. While this suggests that some individuals may place greater importance on the news source than assessing the credibility of the information, the latter point is critical in the context of public health messaging. Responses to such messaging are a key component of the work by Nimbi et al. who focus on the perception of news relating to the recent Monkeypox outbreak. Their findings show that older, heterosexual, politically conservative, and religious individuals were more likely to disregard information about this outbreak as being a hoax (i.e., fake news). Such findings are important as they highlight which sections of society that health professionals, with limited resources, should target with additional health communication strategies and interventions. Finally, in a paper titled “*Noise, Fake News, and Tenacious Bayesians*,” Brody presents an intriguing new modeling framework that incorporates the representation of reliable information, noise, and disinformation, within a unified framework. Findings suggest that the Bayesian framework can be useful in explaining confirmation bias, and that noise can be used as a way of combating the negative impact of disinformation.

As research on fake news and disinformation continues to evolve, we thank the authors who have contributed papers to this special topic. Their novel findings add to the growing body of work that will provide new routes to enhance the detection of fake news, and new interventions to reduce sharing behavior. Taken together, these developments will attenuate the effect of misinformation on the public perception and understanding of news content encountered on social media.

## Author's note

This article provides the introduction to the Frontiers in Psychology special issue on ‘The Psychology of Fake News on Social Media: Who falls for it, who shares it, why, and can we help users detect it?'

## Author contributions

All authors listed have made a substantial, direct, and intellectual contribution to the work and approved it for publication.
